# Identification of an intragenic deletion in the *SGCB* gene through a re-evaluation of negative next generation sequencing results

**DOI:** 10.1016/j.nmd.2016.02.013

**Published:** 2016-06

**Authors:** Teresa Giugliano, Marina Fanin, Marco Savarese, Giulio Piluso, Corrado Angelini, Vincenzo Nigro

**Affiliations:** a Dipartimento di Biochimica, Biofisica e Patologia Generale, Seconda Università di Napoli, Napoli, Italy; b Telethon Institute of Genetics and Medicine, Pozzuoli, Italy; c Dipartimento di Neuroscienze, Università di Padova, Padova, Italy; d Fondazione Ospedale S. Camillo, IRCCS, Venice, Italy

**Keywords:** *SGCB*, Sarcoglycanopathy, LGMD2E, Copy number variation, Deletion, Next generation sequencing

## Abstract

A large mutation screening of 504 patients with muscular dystrophy or myopathy has been performed by next generation sequencing (NGS). Among this cohort of patients, we report a case with a severe form of muscular dystrophy with a proximal weakness in the limb-girdle muscles. Her biopsy revealed typical dystrophic features and immunohistochemistry for α- and γ-sarcoglycans showed an absent reaction, addressing the clinical diagnosis toward a sarcoglycanopathy. Considering that no causative point mutation was detected in any of the four sarcoglycan genes, we re-evaluated the NGS data by careful quantitative analysis of the specific reads mapping on the four sarcoglycan genes. A complete absence of reads from the sixth exon of the β-sarcoglycan gene was found. Subsequent array comparative genomic hybridization (CGH) analysis confirmed the result with the identification of a novel 3.3 kb intragenic deletion in the *SGCB* gene. This case illustrates the importance of a multidisciplinary approach involving clinicians and molecular geneticists and the need for a careful re-evaluation of NGS data.

## Highlights

• 504 myopathic patients have been screened by an NGS approach.
• A patient with a strong suspicion of sarcoglycanopathy, due to WB and immunohistochemical studies, was investigated.
• The absence of reads on the sixth exon of the β-sarcoglycan gene was identified by a careful re-evaluation of the NGS data.
• Subsequent array CGH analysis identified a novel 3.3 kb intragenic deletion in the *SGCB* gene.
• A strong collaboration between clinicians and molecular geneticists is crucial for a careful interpretation of NGS results.

## 1 Introduction

Limb girdle muscular dystrophies (LGMDs) are a large group of heterogeneous autosomal disorders that are somewhat similar, but milder than X-linked Duchenne muscular dystrophy (DMD). As in DMD, a progressive muscle wasting produces an initial weakness of the pelvic and/or shoulder girdle muscles [1].

The sarcoglycanopathies are severe forms of LGMD closely related to the dystrophinopathies. Pathogenic variants in any of the sarcoglycan (SGC) genes produce an imperfect dystrophin- associated SGC complex that fails to localize at the muscle membrane. A stable muscle SGC complex must be heterotetrameric with a 1:1:1:1 ratio between α-, β-, γ- and δ-sarcoglycan with some exceptions in the cardiac and smooth muscles, where ε-sarcoglycan may replace α- sarcoglycan [2], [3], [4]. Sarcoglycanopathies usually have a childhood onset. The quadriceps and posterior thigh muscles are affected together with the shoulders. The progression tends to be more rapid than that of other LGMDs, with a loss of ambulation usually at 12–16 years. Patients with a late onset generally have a slower and more benign course. Cardiomyopathy is reported in about 30% of cases, but it is less common than in LGMD2D. A progressive weakness leads to restrictive lung disease and hypoventilation so that ventilator assistance is often necessary [5], [6]. Cognitive impairment has never been described in patients with sarcoglycanopathy.

In contrast with the Duchenne and Becker muscular dystrophies that are commonly caused by large intragenic deletions/duplications in *DMD*, mutations in SGC genes are typically small defects, such as single nucleotide substitutions or short deletions/insertions. The large size of the *DMD* gene (2200 kb) may partially explain the difference in mutation types. Similarly, a greater number of intragenic deletions/duplications should also be expected in the *SGCG* (144 kb) and the *SGCD* (433 kb) compared with the *SGCA* (10 kb) or the *SGCB* (15 kb) genes.

*SGCG* deletions have been identified by multiplex ligation-dependent probe amplification (MLPA) analysis or targeted array CGH [7], [8], [9], and complete exonic deletions of the *SGCA* gene and partial duplications of different exons of the *SGCB* gene have also been described [6], [10], [11]. Furthermore, in the context of contiguous gene deletion syndrome, a homozygous 400 kb deletion, also comprising the *SGCB* gene, has been mapped by FISH and Southern blot in a large consanguineous East-Anatolian family with an LGMD phenotype [12].

By using MotorPlex, an NGS-based platform for simultaneously testing 93 genes related to primary skeletal muscle diseases [13], we recently performed an extensive mutation screening in a large cohort of 504 patients affected by an LGMD or a myopathy.

In this study, we describe a novel intragenic deletion at the *SGCB* locus, identified by an NGS approach and mapped by an array CGH, associated with a severe LGMD phenotype.

## 2 Case presentation

A female child was born from healthy parents and her family history was negative.

She started presenting a proximal weakness in the limb girdle muscles in early childhood. A neurological examination at the age of 9 years showed a waddling gait, positive Gowers' sign, muscle hypertrophy of the calves and the quadriceps femoris, macroglossia, a severe muscle weakness in the proximal muscles of all four limbs, absent deep tendon reflexes and a high arched palate. Her CK levels ranged from 6549 to 7924 U/L (n.v. 0–190 U/L). EMG showed diffuse myopathic changes.

A quadriceps muscle biopsy showed severe dystrophic changes, consisting of increased fiber size variability, increased central nuclei, opaque fibers, degenerating and regenerating fibers, and marked endomysial fibrosis. Immunohistochemical investigations for α- and γ-sarcoglycan showed an absent reaction.

As a first level genetic test, the patient's genomic DNA was analyzed by using Motorplex, a customized NGS panel for the targeted enrichment of selected genes causing primary skeletal muscle diseases [13]. All the exons and ten intronic flanking bases of the 93 genes included in the MotorPlex design were specifically sequenced on a HiSeq1000 instrument (Illumina, USA).

On average, targeted resequencing generated 4.1 Mb of sequence data as 100-bp paired-end reads. The sequence data were analyzed using an in-house pipeline designed to automate the analysis workflow [14]. About 92.1% of the targeted regions were read more than 100 times, ensuring the detection of genetic variants with a high sensitivity and specificity.

None of rare variants (frequency <0.01) identified in this patient after data filtering appeared to be pathogenetic or in accord with the observed clinical phenotype and the manner of inheritance.

**Fig. 1:**
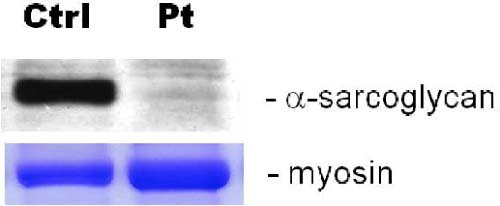
A western blot analysis with different primary antibodies showed the absence of α-sarcoglycan protein.

Meanwhile, a Western blot analysis with different primary antibodies showed the absence of α-sarcoglycan protein (Fig. 1) and a normal expression of the dystrophin, dysferlin, and calpain-3 proteins (data not shown).

**Fig. 2:**
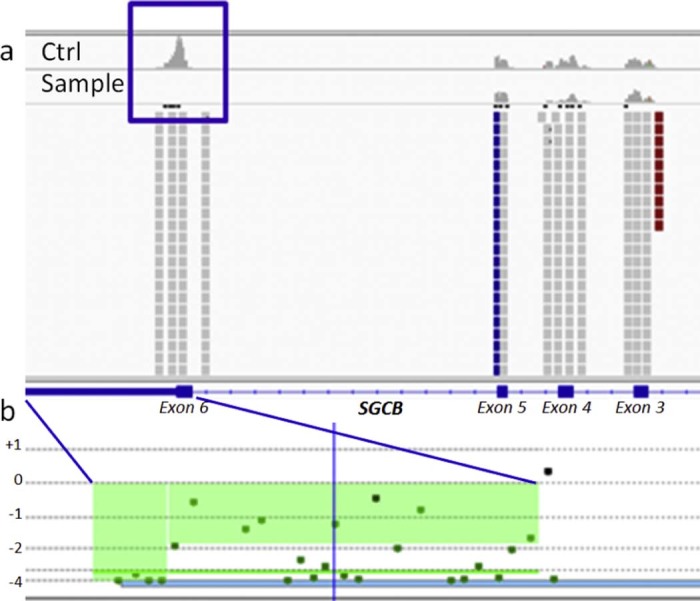
Graphic view of NGS and array CGH results. (a) IGV graphic view of the *SGCB* gene coverage in a control (upper panel) and in the proband sample (lower panel); (b) Motor Chip profile of the last 12 codons of exon 6 and the 3′UTR *SCGB* deletion.

The strong clinical suspicion of sarcoglycanopathy, confirmed by the immunohistochemical and Western blot results, led us to re-evaluate the NGS data, focusing our attention on the *SGC* genes. By checking the coverage obtained by the NGS data for these genes, we discovered the absence of any read on the last exon of the *SGCB* gene, unlike all of the other samples analyzed (Fig. 2a). A homozygous deletion in the last exon of *SGCB* was suspected. The amplification of all the exons showed no PCR products for exon 6 in the patient compared to a control.

To confirm the deletion, we performed array CGH analysis using Motor Chip, an oligonucleotide-based 8X60K microarray (Agilent Technologies, USA) with exon-specific gene coverage in 245 genes involved in neuromuscular disorders, as well as 180 candidate disease genes [7]. Motor Chip was able to further define this novel homozygous deletion that included the last 12 codons of exon 6 and the 3′UTR of the *SGCB* gene, spanning about 3.3 kb at 4q12 (Fig. 2b).

## 3 Discussion

The development of NGS technologies has improved the genetic understanding of various diseases, especially disorders characterized by a genetic and phenotypic heterogeneity.

For LGMDs, where the list of genes to be screened is too large for the gene-by-gene approach, a targeted NGS approach, with panels including all the genes so far associated with these disorders, represents a straightforward strategy [13].

For the patient described here, the NGS analysis was initially negative as no pathogenic variants were identified. However, the strong clinical suspicion of a sarcoglycanopathy directed us to a re-evaluation of the NGS data, by careful quantitative analysis of the specific reads mapping on the four sarcoglycan genes. The absence of reads in the sixth exon of the *SGCB* gene was observed, suggesting a homozygous deletion, subsequently confirmed using Motor Chip for a better definition of this copy number variation (CNV).

CNVs are a rare occurrence in the sarcoglycanopathies [10], [11], [12]. However, the number of CNVs in the *SGC* genes could be underestimated because of the limitations of standard technologies.

To date, the bioinformatics tools for the detection of CNVs from NGS data do not provide a sufficient specificity and sensitivity [15]. Any NGS tool is able to investigate single nucleotide variants but it can only suggest the presence of gene deletions/duplications, as described here. An array CGH analysis should anyway be carried out either to confirm or better characterize putative CNVs suggested by the NGS analysis or to reveal CNVs when no causative mutations have been identified.

Although conventional molecular methods are still important for clinical application, this report highlights the increased role of NGS in routine clinical diagnostics and the need for a careful interpretation of the results by a molecular geneticist. NGS tools are used as a first tier test for most diagnostic centers, despite the fact that they should be a last resort for LGMDs according to the American Academy of Neurology Guidelines [16].

The employment and the cost-effectiveness of the NGS analysis in the LGMD diagnostic workflow are still debatable [17]. A large study comparing patients who had undergone a biopsy first followed by NGS testing versus patients tested with NGS as the first step should be performed to definitively demonstrate the cost- and time-effectiveness of the NGS approach.

Regardless of the effective order of the diagnostic workflow, a multidisciplinary approach involving clinicians and molecular geneticists is crucial for the correct interpretation of data generated by these high throughput technologies, as clearly demonstrated here.
